# Evaluation of treatment-related serological changes in *Treponema pallidum* subsp. *endemicum* infection using latex-based immunoassays, with comparison to *Treponema pallidum* subsp. *pallidum*

**DOI:** 10.1371/journal.pntd.0014756

**Published:** 2026-10-15

**Authors:** Yu-ki Deno, Keiyu Yamamoto, Mie Matsumoto, Toshihiko Fujikawa, Fumiya Banno, Takuya Kawahata, Keiichi Furubayashi

**Affiliations:** 1 Customer Support Center, Diagnostics Division, Sekisui Medical Co., Ltd., Tokyo, Japan; 2 Diagnostic Product Development Department, Research & Development, Sekisui Medical Co., Ltd., Tokyo, Japan; 3 Division of Microbiology, Osaka Institute of Public Health, Osaka, Japan; 4 Sonezaki Furubayashi Clinic, Osaka, Japan; UConn Health, UNITED STATES OF AMERICA

## Abstract

**Background:**

*Treponema pallidum* subsp. *endemicum* (TEN), the causative agent of bejel, is a nonvenereal treponematosis that occurs mainly in arid regions such as Africa and the Middle East. Recently, sexually transmitted TEN infections have been reported outside endemic regions, but their serological characteristics and treatment responses remain unclear.

**Methodology/principal findings:**

We analyzed 6 genetically confirmed bejel patients and 9 syphilis patients in Japan. Serum treponemal (TP) and non-treponemal (RPR) antibody titers were quantified at baseline and post-treatment using latex agglutination test (LA). The analysis of antibody profiles was performed with an immunoblot assay detecting IgM and IgG antibodies against Tp47, TmpA, Tp17, and Tp15. Phylogenetic analysis based on concatenated *tp0548* and *tp0856* sequences identified 6 bejel cases (TEN). Changes in TP and RPR antibody titers following treatment showed no apparent difference between bejel and syphilis groups. In both groups, some IgM antibodies decreased following treatment, while some IgG antibodies increased.

**Conclusions/significance:**

The serological profiles and post-treatment changes in antibody titers were similar between bejel patients and syphilis patients. These findings suggest that conventional serological assays developed for syphilis may also be useful for clinical management of bejel, including evaluation of treatment response. This study provides new insight into the diagnosis and serological monitoring of sexually transmitted bejel cases outside endemic areas.

## Author summary

Bejel, caused by *Treponema pallidum* subsp. *endemicum* (TEN), is one of the endemic treponematoses transmitted mainly through nonsexual contact in arid regions such as the Middle East and the Sahel region of Africa. However, recent reports have identified sexually transmitted TEN infections outside endemic areas. In Japan, we investigated 6 genetically confirmed bejel cases and compared them with 9 cases of *Treponema pallidum* subsp. *pallidum* (TPA) infection, which causes syphilis. Serological testing demonstrated that both bejel and syphilis patients showed similar patterns of treponemal (TP) and non-treponemal (RPR) antibody responses at baseline and post-treatment. Treponemal antibody profiles were comparable between bejel and syphilis patients. These findings suggest that existing serological assays for syphilis may be useful for the clinical management of bejel patients, providing valuable insights into the diagnosis and monitoring of this neglected treponemal disease.

## Introduction

Human pathogenic treponemes include several subspecies such as *Treponema pallidum* subsp. *pallidum* (TPA), *Treponema pallidum* subsp. *pertenue* (TPE), and *Treponema pallidum* subsp. *endemicum* (TEN), which cause syphilis, yaws, and bejel, respectively [1].

Syphilis is a sexually transmitted disease with a worldwide distribution.

In recent years, it has re-emerged as a global public health problem, with increasing incidence over the past decade [2]. In Japan, syphilis incidence has been increasing since 2013, with annual reported cases exceeding 10,000 since 2022 [3,4].

On the other hand, yaws is transmitted through skin contact among children in humid tropical regions. A total of 7,756 cases were reported in 2024 [5]. Despite mass treatment campaigns by the WHO, the disease remains endemic, with increasing incidence in some countries [6].

Bejel is spread in arid regions through skin contact or the sharing of utensils, primarily among children and adolescents. Recently, however, cases among men who have sex with men (MSM) have been reported in France and Cuba [7–9].

Because TPA, TPE, and TEN share 98% nucleotide sequence identity [10], they are difficult to distinguish by serological testing. However, specific treponemal test (TT) and nonspecific nontreponemal test (NTT) for syphilis are used for their diagnosis [11].

Recent advances in molecular diagnostic technology have made it possible to analyze treponemal subspecies and study transmission routes in non-endemic areas [12].

In 2019, we performed PCR testing on patients treated for syphilis and several were identified to be positive for bejel [13]. In 2022, we reported that all identified bejel cases were adult MSM who acquired infection through sexual contact in Japan [7]. As bejel cases have continued to be reported in Japan [14–16], these findings indicate that bejel is present, albeit in small numbers, in non-endemic regions.

For the monitoring of treatment in yaws, point-of-care tests (POCTs) and chemiluminescent antibody assays using whole blood have been developed. These methods have been reported to show good correlation with conventional TPPA and RPR-Card tests in surveys in endemic regions [17–19]. In contrast, although several studies on bejel have been reported [6,20], there have been few systematic investigations, particularly regarding changes in antibody titers before and after treatment on an individual case basis. Accordingly, we hypothesized that the dynamics of treponemal antibodies and non-treponemal antibodies before and after treatment in bejel may be similar to those observed in syphilis.

In this study, we measured antibody titers using a latex agglutination test (LA) [21] and analyzed the variation of antibody species to clarify the differences in serological responses between bejel and syphilis, and to examine the clinical usefulness of existing serological tests.

## Methods

### Ethics approval

The experiments for the identification of TPA and TEN were conducted at the Osaka Institute of Public Health after obtaining approval from its Ethics Review Committee (Approval No. 1812-10-3). Furthermore, the entire study, including the experiments for antibody titer measurement and antibody profiling, was conducted after obtaining approval from the internal ethics review board of Sekisui Medical Co., Ltd. (Approval No. 2019-08).

Written informed consent was obtained from all participants prior to sample collection, and all procedures were performed in accordance with the Declaration of Helsinki.

### Sample selection

Samples were selected according to the flowchart shown in Fig 1.

**Fig 1:**
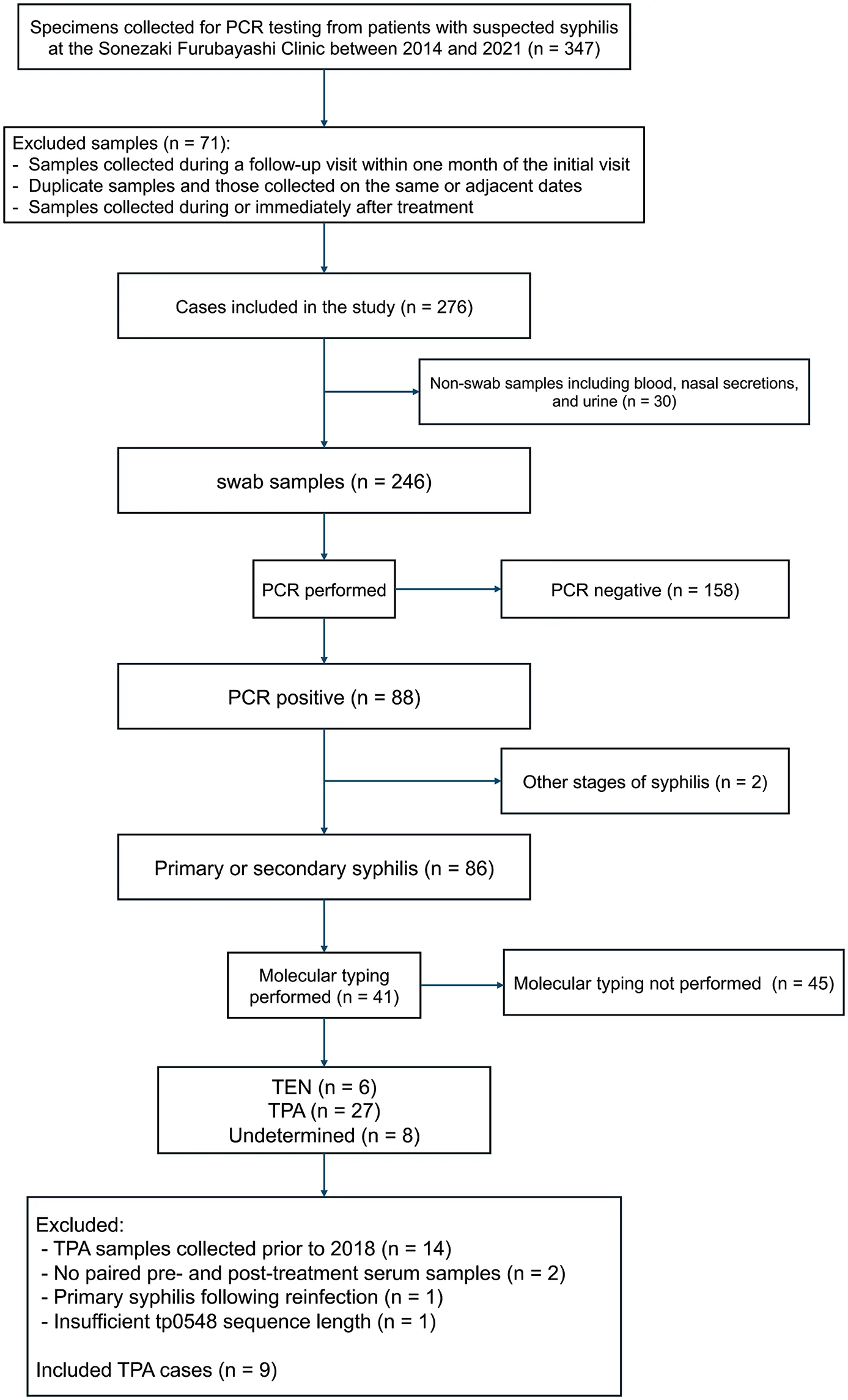
Flow diagram of case selection and specimen processing.

Specimens were collected from patients with suspected syphilis at the Sonezaki Furubayashi Clinic between 2014 and 2021 (n = 347). Of these, 71 samples were excluded because they were either collected during a follow-up visit within one month of the initial visit, were duplicate samples collected on the same or adjacent dates, or were collected during or immediately after treatment, resulting in 276 cases included in the study.

Among these, 246 swab samples were subjected to PCR testing for *Treponema pallidum*, yielding 88 PCR-positive and 158 PCR-negative cases. Of the PCR-positive cases, 86 were clinically classified as primary or secondary syphilis and 2 as other clinical stages.

Molecular typing was performed in 41 cases, identifying 6 *Treponema pallidum* subsp. *endemicum* (TEN), 27 *Treponema pallidum* subsp. *pallidum* (TPA), and 8 undetermined cases, while molecular typing was not performed in 45 cases.

For TPA cases, exclusions included samples collected prior to 2018 (n = 14), lack of paired pre- and post-treatment serum samples (n = 2), primary syphilis following reinfection (n = 1), and insufficient *tp0548* sequence length (n = 1), resulting in 9 TPA cases included in the final serological analysis.

Swab samples collected from patients with suspected syphilis between 2014 and 2021 were subjected to PCR testing for *Treponema pallidum*, followed by molecular typing of PCR-positive cases. Based on the predefined inclusion and exclusion criteria shown in Fig 1, 6 genetically diagnosed TEN cases and 9 TPA cases were included in the final serological analysis. All patients clinically diagnosed with active syphilis received appropriate treatment. Clinical and laboratory data were retrospectively collected from the medical records for cases in which molecular typing identified TEN or TPA. Because of the rarity of bejel and the limited number of eligible cases, this study should be regarded as exploratory, and the results are presented descriptively.

### Sample collection and DNA extraction

Swab samples were collected from lesion sites (e.g., chancres) of patients with suspected syphilis using sterile cotton swabs and suspended in 0.5 mL of phosphate-buffered saline (PBS). Template DNA was extracted from 200 µL of the PBS suspension using the QIAamp DNA Mini Kit (Qiagen, Hilden, Germany), according to the manufacturer’s instructions for use.

### PCR detection of *Treponema pallidum*

Detection of *Treponema pallidum* DNA was performed using PCR targeting the *polA* gene and the TpN47 gene region, as previously described [22,23]. Each 20 µL reaction mixture contained 18 µL of reaction mix and 2 µL of template DNA. PCR amplification was performed under the following conditions: initial denaturation at 95°C for 20 s, followed by 45 cycles of denaturation at 95°C for 1 s and annealing/extension at 60°C for 20 s. Samples were considered positive for *Treponema pallidum* if amplification of either the *polA* gene or the TpN47 gene region was detected.

### Molecular typing of TEN and TPA

PCR products of the *tp0548* and *tp0856* loci from *Treponema pallidum*-positive samples were sequenced using an ABI 3130 genetic analyzer (Thermo Fisher Scientific, Waltham, MA, USA). For subspecies classification, a phylogenetic tree was constructed using concatenated *tp0548* and *tp0856* sequences [24]. When only one locus was successfully amplified, classification was performed based on single-locus phylogenetic analysis together with reference strain sequences.

### Phylogenetic analysis

Sequence alignment was performed using the MUSCLE algorithm implemented in MEGA version 12 [25]. Phylogenetic trees were constructed using the maximum likelihood method with the Tamura–Nei model. The robustness of the inferred trees was evaluated by bootstrap analysis with 1,000 replicates.

### Time of collection of serum samples and antibody assay

Serum samples were collected at 2 time points: at the initial visit and at the end of treatment or upon confirmation of cure. Treponemal antibodies and non-treponemal RPR antibodies were measured using the LA method. Serum samples were stored under refrigeration. After case selection according to the procedure shown in Fig 1, serum samples from eligible patients were simultaneously examined using TPPA, RPR-Card test, and an immunoblot assay.

### Serological tests

Mediace TPLA (Sekisui Medical, Tokyo, Japan; TPLA) and Mediace RPR (Sekisui Medical; RPR-LA) were used as LA reagents. The cutoff values of TPLA and RPR-LA are 10 TPLA UNIT (hereinafter abbreviated as T.U.) and 1.0 RPR UNIT (hereinafter abbreviated as R.U.), respectively. TPPA (Fujirebio, Tokyo, Japan; TPPA) and RPR-Card method (Sekisui Medical; RPR-Card) were used as comparison methods. Their respective cutoff values are ≥1:80 TPPA titer and ≥1:1 RPR-Card method titer, respectively.

### Immunoblot detection of treponemal antibodies

For the analysis of antibody profiles, the recomLine Treponema IgG/IgM (Mikrogen, Neuried, Germany) was used to detect IgG and IgM reactivity to various treponemal antigens. During evaluation, the color intensity of the band was visually assessed and scored using the following criteria: 2 if the intensity was stronger than the control band, 1 if the intensity was lower than the control line, and 0 if no band was observed.

## Results

### Detection and molecular typing of *Treponema pallidum*

Among 41 PCR-positive samples subjected to molecular typing, 6 bejel cases and 9 syphilis cases met the inclusion criteria. Of these, both *tp0548* and *tp0856* sequences were successfully obtained from 5 bejel cases (TEN-1, TEN-2, TEN-3, TEN-4, TEN-5) and 3 syphilis cases (TPA-1, TPA-2, TPA-5) (Fig 2).

**Fig 2:**
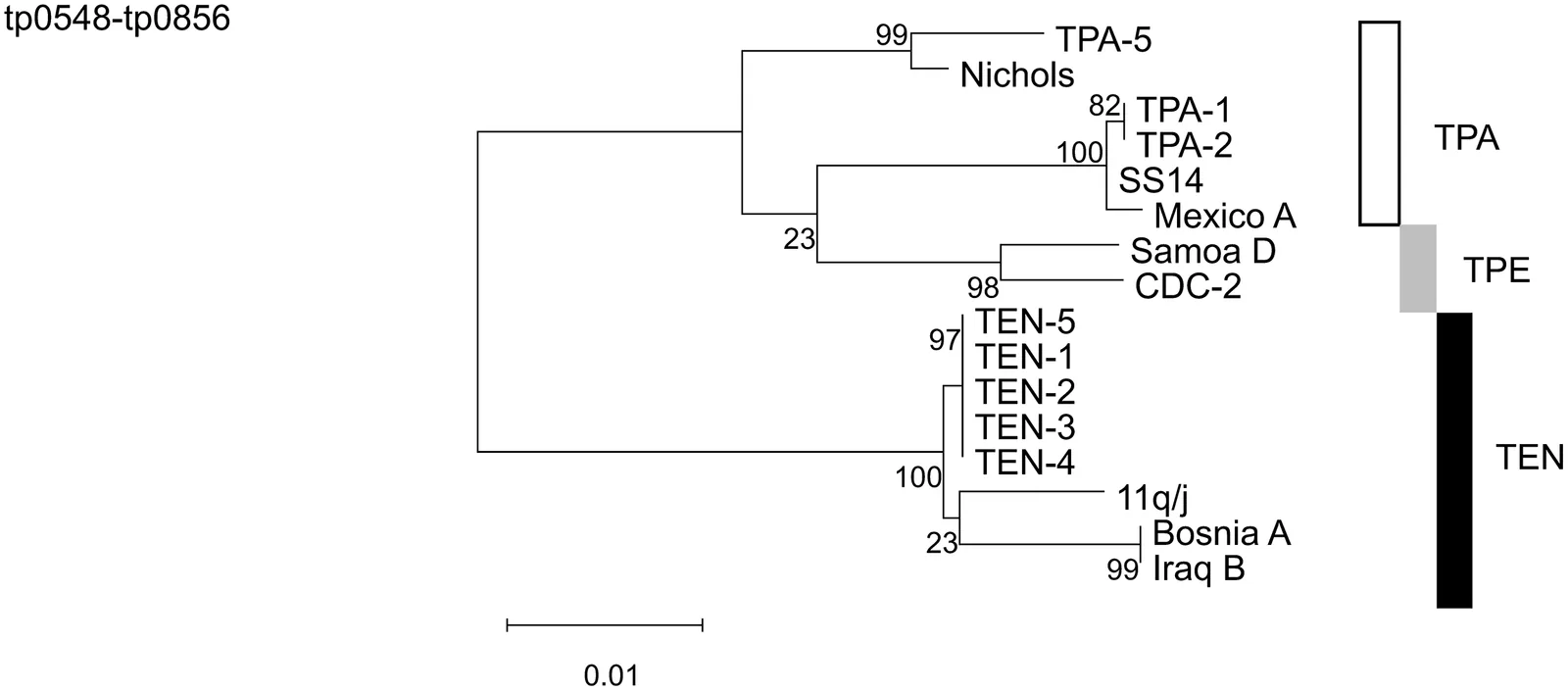
Phylogenetic analysis based on concatenated *tp0548* and *tp0856* sequences. A phylogenetic tree was constructed using concatenated sequences of the *tp0548* and *tp0856* genes from clinical isolates and reference strains. The analyzed sequences included Treponema pallidum subsp. pallidum (TPA), T. pallidum subsp. pertenue (TPE), and T. pallidum subsp. endemicum (TEN). Clinical isolates are indicated as TPA-1 to TPA-9 and TEN-1 to TEN-6. Some isolates were classified based on single-locus sequence data. Reference strains are labeled by their strain names. Bootstrap values (%) are shown at the nodes. The scale bar indicates nucleotide substitutions per site.

For cases in which only a single locus was successfully sequenced, subspecies classification was inferred based on phylogenetic analysis using the available locus together with reference strains. Specifically, 5 cases (TPA-3, TPA-4, TPA-6, TPA-7, and TPA-9) with only *tp0548* sequences clustered with TPA reference strains (S1 Fig). Similarly, TEN-6 and TPA-8, for which only *tp0856* sequences were obtained, clustered with TEN and TPA reference strains, respectively (S2 Fig).

### Trends in antibody titers in bejel and syphilis cases

The clinical characteristics of 6 bejel cases and 9 syphilis cases are shown in Table 1. All 6 bejel cases were male, and 2 bejel cases (TEN-1 and TEN-2) were negative for both TPLA and RPR-LA at the initial visit.

**Table 1:** Clinical features of 6 cases of bejel (TEN) and 9 cases of syphilis (TPA).

Case No.	Age	Sex	Year of consultation	Foreign travel history	HIV- serostatus	Suspected area of acquisition	Presenting clinical symptoms	*tp0548* accession number	*tp0856* accession number	Initial diagnosis at presentation	Initial TPLA titer (T.U.)	Initial RPR-LA titer (R.U.)	Sampling site	Treatment	Outcome
TEN-1*	20	Male	2014	NA	Negative	Osaka	penile ulcer	LC383799	LC430604	bejel (primary lesions)	0.0	0.0	penile ulcer	AMPC 1500 mg/day for 3 weeks	improved following treatment
TEN-2*	24	Male	2017	NA	Negative	NA	penile erosion	LC430602	LC430607	bejel (primary lesions)	4.9	0.0	penile erosion	AMPC 1500 mg/day for 1 week	improved following treatment
TEN-3*	37	Male	2018	NA	On treatment	NA	penile ulcer	LC430603	LC430608	bejel (primary lesions)	32.2	0.1	penile ulcer	AMPC 1500 mg/day for 1 week	improved following treatment
TEN-4	31	Male	2018	France (2015) South America (2016)	Negative	Osaka	hard chancre	LC589188	LC589189	bejel (primary lesions)	46.4	2.5	pharynx penile ulcer	AMPC 1500 mg/day for 1 week and MINO 200mg/day for 3 weeks	improved following treatment
TEN-5*	30	Male	2017	NA	Negative	Osaka	Granulomas at the nasal vestibule, small erythematous macules on the palms, small reddish papules on the mons pubis, small erythematous macules and small ulcers on the penis, and granulomas extending from the perineal to the perianal region	LC383801	LC430605	bejel (secondary lesions)	22672.0	32.0	penile erosion	AMPC 1500 mg/day for 4 weeks	improved following treatment
TEN-6	29	Male	2019	Hawaii (2014) Taiwan (2017)	Negative	Tokyo	roseola-like rash	N.D.	LC589190	bejel (secondary lesions)	11200.0	60.0	pharynx	AMPC 1500 mg/day for 4 weeks	improved following treatment
TPA-1	28	Male	2019	NA	Negative	Osaka	penile ulcer	LC931913	LC931921	primary syphilis	138.6	16.0	penile ulcer	AMPC 1500 mg/day for 4 weeks	improved following treatment
TPA-2	56	Male	2019	NA	Negative	Osaka	penile ulcer	LC931914	LC931922	primary syphilis	13.8	0.2	penile ulcer	AMPC 1500 mg/day for 4 weeks	improved following treatment
TPA-3	43	Male	2019	NA	Negative	NA	penile ulcer	LC931915	N.D.	primary syphilis	14.9	0.5	penile ulcer	AMPC 1500 mg/day for 4 weeks	improved following treatment
TPA-4	50	Male	2019	NA	Negative	NA	penile ulcer	LC931916	N.D.	primary syphilis	151.2	16.0	penile ulcer	AMPC 1500 mg/day for 4 weeks	improved following treatment
TPA-5	66	Male	2021	NA	Negative	NA	penile ulcer	LC931917	LC931923	primary syphilis	19.7	0.0	penile ulcer	AMPC 1500 mg/day for 4 weeks	improved following treatment
TPA-6	61	Male	2021	NA	Negative	Kobe	penile ulcer	LC931918	N.D.	primary syphilis	21.1	0.9	penile ulcer	AMPC 1500 mg/day for 4 weeks	improved following treatment
TPA-7	28	Female	2021	NA	Negative	Osaka	vulvar ulcer	LC931919	N.D.	primary syphilis	45.3	25.0	vulvar ulcer	MINO 200 mg/day for 4 weeks	improved following treatment
TPA-8	32	Male	2019	NA	Negative	Osaka	Erythematous lesions on the trunk, penile papules, and a scrotal ulcer	N.D.	LC931924	secondary syphilis	27616.0	62.0	pharynx	AMPC 1500 mg/day for 4 weeks	improved following treatment
TPA-9	21	Female	2020	NA	Negative	Osaka	Erythematous lesions on the palms, soles, and trunk, and genital erosion	LC931920	N.D.	secondary syphilis	5912.0	30.0	genital erosion	MINO 200 mg/day for 4 weeks	improved following treatment

Bejel cases were categorized based on lesion characteristics corresponding to stages of syphilis.

Abbreviation: T.U., TPLA units; R.U., RPR units; AMPC, amoxicillin; MINO, minocycline; NA, not available; N.D., not determined.

*The specimens TEN-1, 2, 3, and 5 were identical to those analyzed in the study by Shinohara et al. [7]

TEN-3 was negative for RPR-LA and a low TPLA titer. TPLA was positive for all syphilis cases, but 4 of 9 cases were negative for RPR-LA and had a low TPLA titer. Clinical symptoms improved in all cases from the initial visit to 30 days after the end of treatment, and treatment was considered complete.

TPPA and RPR-Card testing were performed. Although some discrepancies were observed in high-titer samples, the coefficient of determination (R^2^) ranged from 0.85–0.97 between TPPA and TPLA and 0.86–0.89 between RPR-Card and RPR-LA for bejel, syphilis, and all cases (Table 2).

**Table 2:** Correlation between TPPA and TPLA assay values and between RPR card and RPR-LA assay values.

Assay	Comparison	Sample	N	Regression equation	R^2^
TPLA	vs. TPPA	ALL	29	y = 0.37x - 68.38	0.91
		bejel (TEN)	11	y = 0.43x -131.03	0.85
		syphilis (TPA)	18	y = 0.34x - 236.05	0.97
RPR-LA	vs. RPR-Card	ALL	30	y = 1.14x + 1.35	0.86
		bejel (TEN)	12	y = 1.44x - 0.05	0.89
		syphilis (TPA)	18	y = 1.02x + 2.08	0.89

Separate regression analyses for the pre-treatment and post-treatment subgroups are presented in S1 Table.

Fig 3 shows the changes in TPLA and RPR-LA values before and after

**Fig 3:**
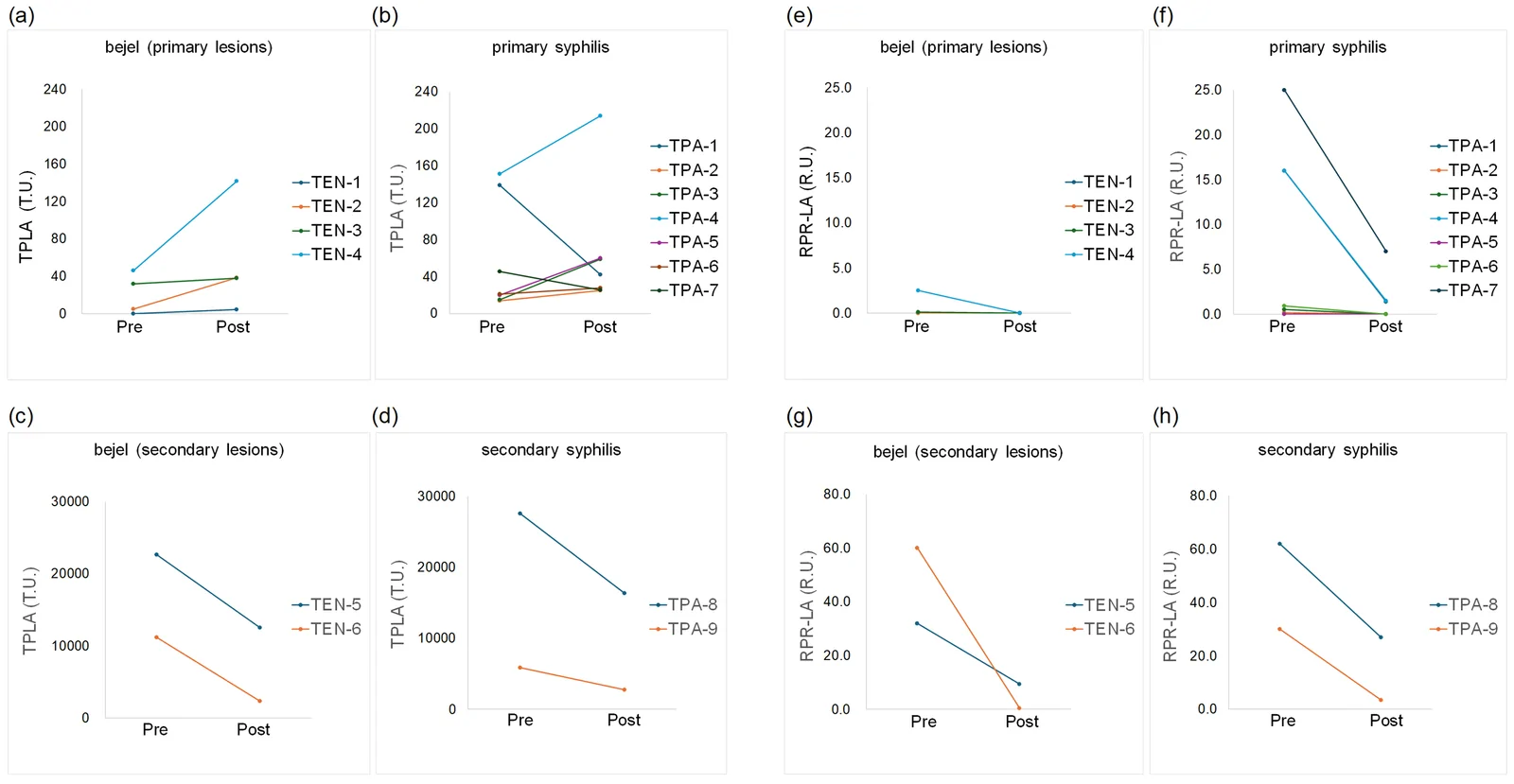
Changes in TPLA and RPR-LA values before and after treatment. **(a–d)** Changes in TPLA values. **(e–h)** Changes in RPR-LA values. **(a, c, e, g)**: bejel cases. **(b, d, f, h)**: syphilis cases. Abbreviation: Pre, pre-treatment; Post, post-treatment; T.U., TPLA units; R.U., RPR units.

treatment according to disease stage. The corresponding individual serological and immunoblot data are provided in S2 Table. TPLA values increased after treatment in 3 of 4 bejel cases with primary lesions (TEN-2, TEN-3, TEN-4) and in 5 of 7 primary syphilis cases (TPA-2, TPA-3, TPA-4, TPA-5, TPA-6) (Table 1). In TEN-1, although TPLA values increased after treatment, no increase was considered to have occurred because both pre- and post-treatment values were below the quantitative range. In all 4 bejel cases with primary lesions and 7 primary syphilis cases, RPR-LA values decreased or remained negative after treatment.

Fig 4 summarizes the changes in treponemal antibodies to specific

**Fig 4:**
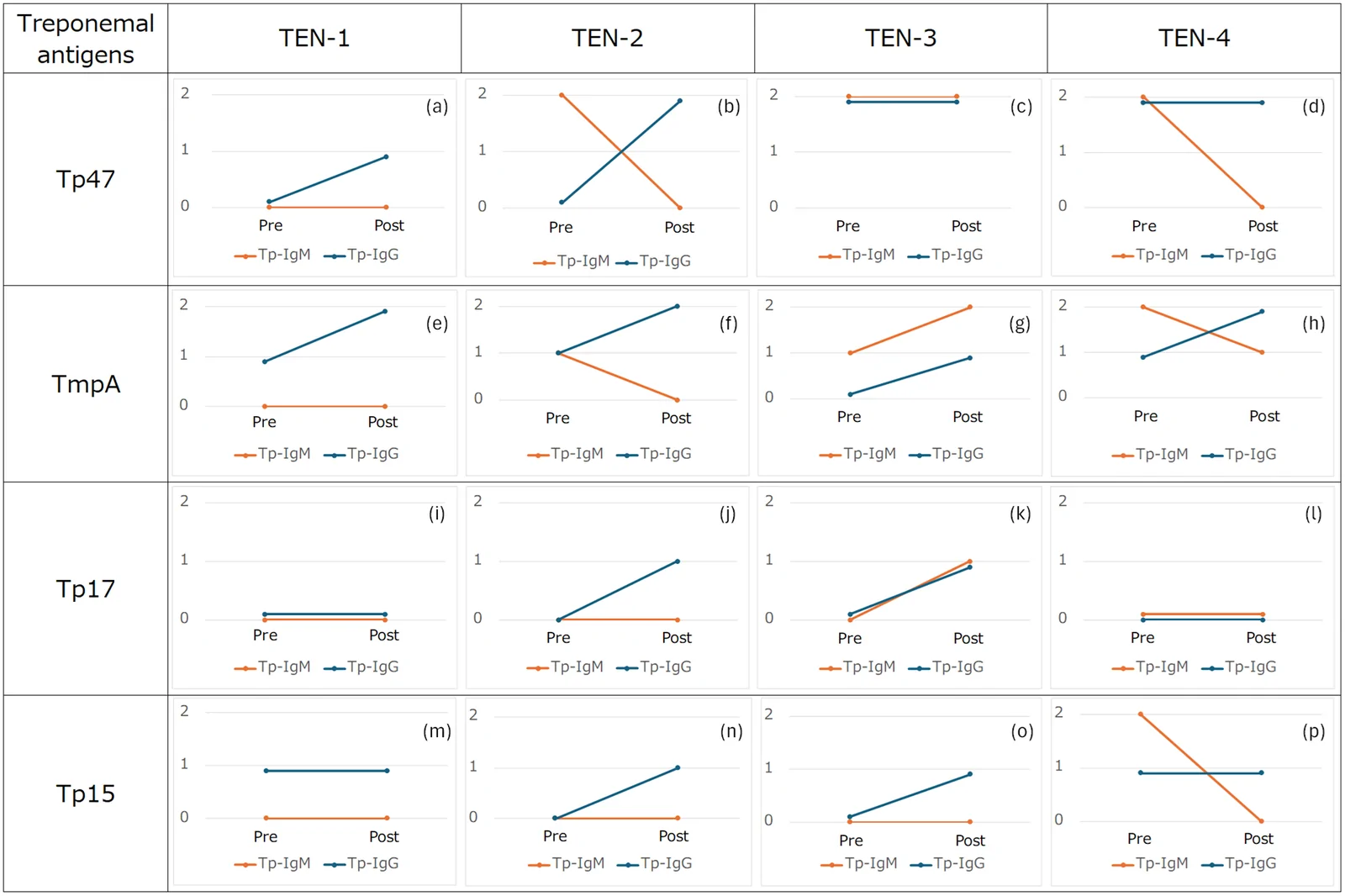
Changes in treponemal antibody activity before and after treatment in bejel cases. The y-axis indicates antibody detection scores (IgM and IgG). Each panel represents an individual case. Abbreviations: Tp-IgM, IgM detection score; Tp-IgG, IgG detection score; Pre, pre-treatment; Post, post-treatment.

antigens in 4 bejel cases with primary lesions. In TEN-2 and TEN-3, TPLA values increased after treatment. In TEN-2, IgM reactivity decreased for Tp47 and TmpA, while IgG reactivity increased for Tp47 and TmpA (Fig 4 (b), (f)). In TEN-4, IgM reactivity decreased for Tp47 and TmpA, while IgG reactivity increased for TmpA (Fig 4 (h)). In TEN-3, no clear decrease in IgM reactivity with a corresponding increase in IgG reactivity against the same antigen was observed. However, an increase in IgG reactivity against Tp15 was detected after treatment (Fig 4 (p)). In the remaining 2 cases in which TPLA values did not increase, no simultaneous decrease in IgM reactivity and increase in IgG reactivity was observed.

In syphilis cases, IgM reactivity decreased and IgG reactivity increased from before to after treatment for Tp47 and TmpA in TPA-2, Tp15 in TPA-3, TmpA, Tp17, and Tp15 in TPA-4, Tp47 in TPA-5, and Tp47 and TmpA in TPA-6 (Table 1). Among syphilis cases in which TPLA values increased after treatment, a decrease in IgM reactivity with a corresponding increase in IgG reactivity was observed for at least one of the four major antigens.

In 2 bejel cases with secondary lesions and 2 cases of secondary syphilis, both TPLA and RPR values decreased rapidly after treatment. In addition, no significant change in antibody reactivity was observed.

## Discussion

In this study, antibody titers were measured before and after treatment in bejel and syphilis cases identified by molecular typing using both LA-based and conventional serological methods, and changes in Treponemal antibody reactivity patterns were analyzed. A strong coefficient of determination was observed between the LA and conventional methods in both bejel and syphilis cases, consistent with previous reports, including those by the present authors [21]. These results suggest that changes in antibody titers measured by TPPA and RPR-Card can be quantitatively evaluated using TPLA and RPR-LA.

A transient increase in TPLA and a decrease in RPR were observed after treatment in 5 of 7 cases for primary syphilis (Fig 3). These changes were associated with changes in antibody classes, including a decrease in IgM and an increase in IgG (Fig 4). Similar changes in antibody titers were observed in 3 of 4 bejel cases with primary lesions (Fig 3), and similar trends were observed in antibody reactivity patterns (Fig 4). These findings suggest that bejel may exhibit serological response patterns similar to those observed in syphilis.

In addition, cases were observed in which both TPLA and RPR-LA values were below the cutoff value at the initial visit, as well as cases that were positive for TPLA but negative for RPR-LA. However, all of these patients presented with clinical symptoms, necessitating appropriate therapeutic intervention. Although serological tests may be negative in a certain proportion of PCR-positive patients [26], it is not practical to perform PCR testing in all cases. Therefore, it is important to evaluate patients by integrating clinical findings with quantitative serological testing.

In the comparison between bejel and syphilis, slight differences were observed in the slopes and intercepts of the regression lines of TPLA and RPR-LA against TPPA and the RPR-Card test (Table 2). These findings suggest that, for the detection of bejel, further evaluation with a larger number of cases may be required rather than directly applying the cutoff values established for syphilis.

In recent years, the possibility of bejel transmission through sexual contact has been reported [7–9], and its presence is becoming a growing clinical and public health concern even in non-endemic regions. Because bejel is clinically and serologically similar to syphilis, there is a risk of misdiagnosis.

Although immunoassays using the LA method require laboratory infrastructure and are therefore limited in resource-constrained settings, they have advantages in terms of quantitative capability, objectivity, and high-throughput processing. In Japan, these assays are widely used in routine medical practice and prenatal screening and are useful for monitoring epidemiological trends in syphilis infection [27]. Accurate differentiation between bejel and syphilis requires further development of molecular diagnostic methods. Meanwhile, quantitative serological testing may contribute to the recognition of atypical cases and to a better understanding of infection dynamics.

The findings of this exploratory study suggest that both treponemal and non-treponemal antibodies can be detected in bejel using conventional serological tests. Although these tests cannot clearly distinguish bejel from syphilis, they may be useful for clinical management, including the evaluation of therapeutic response, particularly in non-endemic regions.

This study has several limitations. First, the number of cases was small and limited to patients in Japan. Second, the timing of infection, diagnosis, and treatment courses were not uniform, and treatment regimens were not standardized. Therefore, the generalizability of the findings is limited, and further studies in larger and more diverse populations are needed. Despite these limitations, our findings provide a basis for further investigation of bejel in non-endemic regions. Accordingly, these findings should be interpreted cautiously because of the exploratory nature of the data, and confirmation in larger studies is warranted.

## Conclusion

Our findings from this exploratory study suggest that Mediace TPLA and Mediace RPR can be used to evaluate serological responses in bejel, with antibody dynamics that appear similar to those observed in syphilis.

## Supporting information

S1 Fig Phylogenetic analysis based on *tp0548* sequences. A phylogenetic tree was constructed using *tp0548* sequences from clinical isolates and reference strains. This single-locus analysis was performed to support subspecies classification in cases where only *tp0548* sequences were available. Bootstrap values (%) are shown at the nodes. The scale bar indicates nucleotide substitutions per site.
(TIF)

S2 Fig Phylogenetic analysis based on *tp0856* sequences. A phylogenetic tree was constructed using *tp0856* sequences from clinical isolates and reference strains. This single-locus analysis was performed to support subspecies classification in cases where only *tp0856* sequences were available. Bootstrap values (%) are shown at the nodes. The scale bar indicates nucleotide substitutions per site.
(TIF)

S1 Table Regression analyses of TPPA versus TPLA and RPR-Card versus RPR-LA by disease group and sampling time. Regression equations and coefficients of determination (R²) are shown for all samples, bejel (TEN), syphilis (TPA), and the pre-treatment and post-treatment subgroups. Because of the limited number of cases in each subgroup, these regression analyses should be interpreted as exploratory. Abbreviations: TPPA, *Treponema pallidum* particle agglutination assay; TPLA, latex agglutination assay for *Treponema pallidum* antibodies; RPR, rapid plasma reagin; RPR-LA, latex agglutination assay for rapid plasma reagin.
(XLSX)

S2 Table Results of each assay and temporal changes in anti-treponemal antibody types in bejel cases and syphilis cases. Late lesions refer to lesions observed after treatment or at later clinical stages. Abbreviations: N.T., not tested; T.U., TPLA units; R.U., RPR units.
(XLSX)
