# Supplementary material for: Evaluation of treatment-related serological changes in Treponema pallidum subsp. endemicum infection using latex-based immunoassays, with comparison to Treponema pallidum subsp. pallidum
Source: PLoS Negl Trop Dis. 2026 Oct 15;20(10):e0014756. doi: 10.1371/journal.pntd.0014756 (PMC13671511; doi:10.1371/journal.pntd.0014756)
Supplement: Supplementary file 1 [file pntd.0014756.s005.pdf]

Dear Reviewer,

Thank you very much for reviewing our paper. Based on your helpful comments, we have made substantial revisions to improve the structure to clarify aspects of our analysis and conclusion. We have added four syphilis cases, however, no additional data were obtained from these cases. Please find below the summary of the major changes.

1. Title has been revised.
Initial posting: "Serological characteristics and treatment-related changes in *Treponema pallidum* subsp. *endemicum* infection: Comparison with *T. pallidum* subsp. *pallidum*."
In this post: "Evaluation of treatment-related serological changes in *Treponema pallidum* subsp. *endemicum* infection using latex-based immunoassays, with comparison to *Treponema pallidum* subsp. *Pallidum*"

2. The procedure for selecting cases was summarized in a flowchart (Fig 1).

3. The background of each case (Age, sex, clinical presentation, and treatment) was summarized in detail.
Four syphilis cases were added, resulting in a total of 6 bejel cases and 9 syphilis cases.
Case NOs are now numbered according to the chronological order of the first visit (Table 1).

4. A phylogenetic tree of the cases was added (Fig 2).

5. The correlation between the latex method and the reference method (TPPA, RPR-Card) was confirmed (Table 2).

6. Because the timing and frequency of serum testing varied among cases, two representative time points were selected for comparison: initial diagnosis (diagnosis and initiation of treatment) and 30 days after completion of treatment (resolution of clinical symptoms) (Fig 3).

7. Because the number of bejel and syphilis cases were small, statistical analysis was not performed. Instead, the results are presented as a comparison of antibody reactivity patterns (Fig.4) with that of the LA method (Fig 3).

8. The limitation of the small number of cases was acknowledged, and have revised the hypothesis to indicate that the findings are suggestive.

9. Detailed data are provided in the separate figures and tables (S1,2 Fig, Tab.2).

10. The medical and public health importance of this study, including its relevance to monitoring sexually transmitted diseases and avoiding misdiagnosis, has been addressed in the Discussion.

Responses to the reviewers' comments are summarized in the table.

We sincerely thank the reviewers for their valuable comments, which have helped us revise the manuscript to improve its clarity and overall quality.

We apologize for any inconvenience caused and appreciate your careful consideration of our work.

NO	Section1	Section2	Reviewer/ Journal	Comment/Question	Answer
1	General		Journal	If the reviewer comments include a recommendation to cite specific previously published works, please review and evaluate these publications to determine whether they are relevant and should be cited. There is no requirement to cite these works unless the editor has indicated otherwise.	-
2			Journal	・Does the analysis presented match the analysis plan?<br>・Are the results clearly and completely presented?<br>・Are the figures (Tables, Images) of sufficient quality for clarity?	-
3	Results		2	・The results are presented clearly and in sufficient detail, and the tables and figures provide useful information to understand the serological dynamics observed in the study population.	-
4	Results		2	• Clarifications are needed regarding Table 1:<br>o TEN Case 1:<br>Why is there a second visit labeled “TEN infection, before treatment”?<br>Please clarify whether treatment was not initiated immediately after the first visit or whether there was a delay between diagnosis and treatment initiation.<br>o TEN Case 2:<br>Why is the first visit labeled “TEN infection, in treatment” and the second visit still described as “TEN infection, after treatment” rather than “Resolved”?<br>Clarification of the clinical status terminology used in the table would improve interpretability.	We appreciate this important comment. All enrolled patients who were clinically diagnosed as having a high likelihood of active syphilis promptly received appropriate treatment after diagnosis. In the revised manuscript, terminology describing treatment timing and clinical status in Table 1 was reorganized and simplified to avoid ambiguity. Terms such as “before treatment” and “after treatment” used in the original version were removed because the retrospective nature of this study did not allow strict standardization of sample collection timing.
5	Results		2	• Tables and figures:<br>o Tables 1 and 2 are detailed and provide essential information on the clinical findings and antibody profiles of the included cases.<br>o Table 2 and Figures 2, 3, and 4 appear to present partially overlapping or complementary information.<br>The authors may consider moving some of these figures to the supplementary material to improve the readability and flow of the main manuscript.	The manuscript has been revised in accordance with your comment.
6	Results		2	• Figure 1:<br>o Figure 1, which presents the changes in antibody titers over time, is currently very dense and difficult to interpret visually.<br>o Suggestion for improvement:<br>▪ Standardize the axis scales across panels, and<br>▪ Consider grouping the graphs more clearly to facilitate immediate visual comparison between TPA and TEN cases or primary vs secondary.	Figure 3 (formerly Figure 1) was reorganized to improve readability and facilitate comparison between bejel and syphilis cases, as well as between primary and secondary lesions. Axis scales and panel grouping were standardized accordingly.
7	Results		2	• Statistical analysis:<br>o The manuscript reports that a two-tailed Welch’s t-test was used to compare the rates of change in antibody titers.<br>o Given the very small sample size ($n < 30$), the authors should clarify:<br>▪ whether the normality of the data distribution was assessed, and<br>▪ whether a non-parametric test (e.g., Mann–Whitney U test) might provide a more robust comparison under these conditions.	Due to the small number of cases, statistical analyses were removed from the revised manuscript. Based on the observed changes in antibody titers and antibody profiles, the conclusions were revised to indicate only possible similarities between bejel and syphilis serological responses.
8	Results		3	1- The authors did not provide any phylogenetic analysis; without a reference phylogeny showing how their isolates cluster relative to established TPA and TEN lineages, their proposed subspecies assignments remain unsubstantiated.	Phylogenetic analyses have been added and are presented in Fig. 2, Supplementary Fig. 1, and Supplementary Fig. 2.
9	Results		3	2-In general, the quality of the figures needs to be improved; all axes should be clearly and consistently labeled to ensure the data can be interpreted accurately.	Figure quality and axis labeling were revised throughout the manuscript to improve clarity and consistency.
10	Summary and General Comments		Journal Requirements	Use this section to provide overall comments, discuss strengths/weaknesses of the study, novelty, significance, general execution and scholarship. You may also include additional comments for the author, including concerns about dual publication, research ethics, or publication ethics. If requesting major revision, please articulate the new experiments that are needed.	-
11			1	This is a straightforward paper. The results are I think unsurprising given the existing literature but there are few recent papers on endemicum espcially in high income settings.<br>The introduction reads as if we dont really know anything about the performance of serology in the endemic treponematoses but of course the performance of these tests in yaws including both treponemal and non-treponemal tests is extremely well described including for monitoring response to treatment - there are many papers on this. I think it would be helpful to incorporate this because the a priori position I think would be to anticipate that serology performs identically between TPE/TPA/TPP. Similarly the discussion totally omits any discussion of what we already know about antibody responses in non-venereal trepoentmatoses and needs to be re-written to incorporate that knowledge.	The manuscript has been revised in accordance with your comment.
12			1	Please try and use person centred language and avoid describing people as 'TEN infected' etc.	Terminology throughout the manuscript was revised to use person-centered language.
13			1	Please describe in the methods the timing of sample collection - i.e was followup at standard time points or at the discretion of the treating clinicians? The statistical comparisons between groups arent really described in the methods. If you are going to present a comparison please make sure that is specified in the methods.	Baseline samples were collected at the initial visit.<br>Follow-up evaluations and specimen collection were performed according to physician instructions at least 30 days after completion of medication.
14			1	I think most readers will find the way the RPR titres are described confusing - did you not get standard titres i.e 1:1, 1:2, 1:4 etc? I think clinical readers will want to understand the fall in terms of dilutions over time not units per day.	We have added correlation data between the standard TPPA, the RPR card test, and the latex agglutination method. As the correlations between these methods were found to be good, we believe this minimizes potential confusion for readers.
15			1	In general I think the results are too long for what is essentially a small comparison of 2 groups of about 5 patients each. They could be greatly simplified to describe baseline and endline titres with 1-2 tables or figures maximum.	To improve readability and organization, the data were reorganized into main and supplementary figures and tables. We also simplified portions of the Results section and clarified the presentation of longitudinal serological changes.
16			2	• This manuscript examines the serological characteristics and treatment-related changes in Treponema pallidum subsp. endemicum (TEN) infection and compares them with those observed in Treponema pallidum subsp. pallidum (TPA) infection. The topic is timely and relevant, particularly in light of recent reports of sexually transmitted TEN infections outside traditionally endemic regions.	Thank you for your comment.

17			2	• The study addresses an important diagnostic and epidemiological question: whether conventional serological assays developed for syphilis can be applied to TEN infection and whether antibody kinetics after treatment behave similarly in both infections. This issue has practical implications for clinical diagnosis, laboratory interpretation of serology, and surveillance systems, particularly in non-endemic settings where TEN infections may be misclassified as syphilis.	Thank you for your comment.
18			2	• The manuscript has several notable strengths:<br>o The study includes genetically confirmed TEN cases, which are rarely reported in the literature.<br>o The authors combine molecular identification with serological and immunoblot analyses, providing a multidimensional characterization of the infections.<br>o The comparison between TEN and TPA infections is conceptually relevant, especially in the context of emerging sexually transmitted TEN cases.<br>o The manuscript provides detailed individual-level serological data, which may contribute valuable preliminary information to a limited evidence base.	Thank you for your positive and encouraging comments.
19		1. Major methodological limitations	2	• The sample size is extremely small (n = 11; 6 TEN and 5 TPA cases). While the rarity of sexually transmitted TEN infections is acknowledged, this limited sample size substantially restricts the statistical power and generalizability of the findings.	As the reviewer pointed out, the sample size was too small to apply statistical methods; therefore, we revised the manuscript to present the results on a case-by-case basis.
20			2	• The manuscript currently applies inferential statistical tests (Welch's t-test) despite the very small sample size and the exploratory nature of the dataset. The authors should clarify whether the study should be considered exploratory/descriptive rather than hypothesis-testing.	As the reviewer pointed out, the sample size was too small to apply statistical methods; therefore, we revised the manuscript to present the results on a case-by-case basis.
21			2	• The study design and patient selection process are not sufficiently described. The manuscript should clarify:<br>o the recruitment period,<br>o the selection criteria for inclusion,<br>o whether the TEN cases represent all identified cases during the study period, and<br>o how the TPA comparator cases were selected.	The relevant information is presented in the Methods section and Figure 1.
22			2	• The clinical characterization of the patients is limited. Additional information regarding age, sex, HIV status, comorbidities, and stage of infection would improve interpretation of the serological findings.	Additional information has been included in Table 1.
23			2	• The treatment regimens administered and treatment adherence are not described, yet treatment could directly influence serological kinetics.	Additional information has been included in Table 1.
24			2	• The follow-up intervals were highly variable (31–515 days), and in some cases the recommended follow-up period for evaluating serological decline after treatment was not completed. This variability should be acknowledged as a limitation.	The analysis was limited to two time points: the initial visit (day 0) and post-treatment follow-up (30 to 189 days after completion of treatment).
25		2. Interpretation of findings	2	• The laboratory methods require clarification, particularly regarding:<br>o the clinical sample type used for DNA extraction, and<br>o the criteria used to classify cases as TEN versus TPA when sequence data were incomplete.	The methodology has been described in more detail.
26			2	• The authors conclude that serological assays developed for syphilis may be applicable to TEN infection. While the data suggest that similar antibody patterns may occur, the current evidence should be interpreted cautiously due to the small sample size and exploratory design.	The discussion section has been revised in accordance with your comment.
27			2	• The conclusions would benefit from more cautious wording. For example:<br>"Our findings suggest that serological responses in TEN infection may resemble those observed in TPA infection."	We have revised the conslusion to indicate the findings are suggestive as we considered that further detailed investigation is necessary.
28			2	• The manuscript would also benefit from a more detailed discussion of the broader clinical and public health implications, including:<br>o the emergence of sexually transmitted TEN infections,<br>o the diagnostic challenges in non-endemic settings, and<br>o the potential misclassification of TEN infection as syphilis in surveillance systems.	The discussion section has been revised in accordance with your comment.
29		3. Data presentation	2	• The results are generally presented clearly, but some figures and tables could be improved to enhance readability. For example:<br>o Figure 1 is dense and difficult to interpret due to multiple panels with differing axes.<br>o Summary statistics (e.g., medians and interquartile ranges) could complement the individual-level trajectories.<br>o Some figures could be moved to supplementary material to streamline the main text.	The manuscript has been revised in accordance with your comment.
30	Recommendation		2	• Overall, this manuscript addresses a relevant and underexplored topic and provides preliminary clinical and serological observations on TEN infections in a non-endemic setting. However, the methodological description, interpretation of findings, and discussion of limitations require substantial clarification.	The manuscript has been revised in accordance with your comment.
31	Recommendation		2	• For these reasons, minor revision is recommended.<br>• The revision should focus on:<br>o clarifying the study design and patient selection,<br>o improving the description of the study population and treatments,<br>o moderating the interpretation of the findings,<br>o strengthening the discussion of limitations and public health relevance, and<br>o improving the presentation of the results and figures.	The manuscript has been revised in accordance with your comment.
32	Recommendation		3	This study addresses an interesting and clinically relevant topic. Understanding how treponemal versus nontreponemal serology behaves across T. pallidum subspecies and how titers evolve post-treatment would substantially inform diagnostics and patient management. However, Deno et al. do not provide the statistical rigor required to support their central claim: "The serological profiles and post-treatment changes in TEN-infected patients were similar to those observed in TPA-infected patients."	As noted above, formal statistical analyses were removed because of the limited sample size, and the conclusions were revised to reflect only exploratory observations.

33	Major concerns:		3	1- Severe underpowering precludes equivalence claims<br>With n=6 TEN vs n=5 TPA, the study can only detect extremely large between-group differences. A non-significant Welch t-test ($p > 0.05$) may reflect this low power rather than evidence of equivalence. This could be the classic Type II error risk and non-significant results cannot substantiate "similarity." If the authors intend to claim practical equivalence (i.e., differences are clinically negligible), they could use methods like equivalence testing (e.g., TOST) with a priori justified equivalence bounds (SESOI) and demonstrate that the 90% CI for the group difference falls entirely within those bounds.<br>The TP/day and RPR/day analyses are particularly fragile given extreme outliers (e.g. -361.8, -218.6 T.U./day), marked skewness, and high variability (SDs around 100-160 for T.U./day). These properties violate parametric t-test assumptions and render confidence intervals uninformative. The non-significant results should be framed as inconclusive. Authors should:<br>a- Reframe as exploratory analyses<br>b- Report effect sizes (Cohen's d) with 95% CIs<br>c- Present medians/IQRs alongside means<br>d- Consider non-parametric alternatives (Mann-Whitney on change scores)	As noted above, formal statistical analyses were removed because of the limited sample size, and the conclusions were revised to reflect only exploratory observations.
34	Major concerns:		3	2- tp0548/tp0856 sequencing lacks validation and documentation:<br>Phylogeny based on tp0548 and tp0856 is not an established method for TPE/TEN discrimination. The authors fail to justify bypassing the validated 7-locus MLST scheme (Pal-Diaz et al., 2025) or gold-standard whole-genome sequencing. Methodological transparency is also inadequate and no details are provided on sequencing platform (Sanger vs. NGS), bioinformatics pipeline (reference genome, variant calling), or phylogenetic context.	The methodology has been revised.
35	Methods		Journal	・Are the objectives of the study clearly articulated with a clear testable hypothesis stated?<br>・Is the study design appropriate to address the stated objectives?<br>・Is the population clearly described and appropriate for the hypothesis being tested?<br>・Is the sample size sufficient to ensure adequate power to address the hypothesis being tested?<br>・Were correct statistical analysis used to support conclusions?<br>・Are there concerns about ethical or regulatory requirements being met?	-
36			2	• The study is described as having an observational, prospective, and analytical design, which allows comparison of the serological behavior between patients infected with Treponema pallidum subsp. pallidum (TPA) and Treponema pallidum subsp. endemicum (TEN). However, the manuscript should explicitly state the study design early in the Methods section (e.g., prospective observational cohort or prospective case series with comparative analysis).	This is a retrospective study.<br>Among the enrolled cases, all patients who were clinically diagnosed as having a high likelihood of active syphilis promptly received appropriate treatment.<br>Data were retrospectively collected and analyzed from the medical records of cases in which molecular typing of the collected specimens identified either TEN or TPA. This information has been added to the Methods section.
37			2	• The study setting is insufficiently described. The authors should clearly specify:<br>• the clinical setting at the institutions involved,<br>• the time period of recruitment,<br>• It remains unclear whether the samples were originally collected for routine clinical care or for research purposes. Clarification is required to understand the context of sample collection and potential selection bias.	The methodology has been described in more detail.
38			2	• Eligibility criteria and participant selection should be described in greater detail. Specifically, the manuscript should report:<br>• the inclusion and exclusion criteria,<br>• the sources of participant recruitment,<br>• the methods used for participant selection, and<br>• the procedures used for follow-up assessments.	The methodology has been described in more detail.
39			2	• The study population is extremely small (6 TEN cases and 5 TPA cases). While it is acknowledged that sexually transmitted TEN in non-endemic settings is rare, the authors should address whether this sample size is sufficient to ensure adequate statistical power to detect meaningful differences between groups.	Given the small sample size, statistical comparisons were omitted, and the conclusions were limited to indicating possible trends based on the observed findings.
40			2	• In its current form, the manuscript does not include a sample size calculation or power estimation. The authors should either:<br>• provide a power calculation, or<br>• explicitly acknowledge that the study should be interpreted as exploratory or descriptive.	We have added clarification that this study should be interpreted as exploratory and descriptive in nature.
41			2	• The representativeness of the included cases should be justified. In particular, the authors should clarify whether these 11 cases represent:<br>• all identified cases during the study period, or<br>• a selected subset.<br>This clarification is important in the context of the recent epidemiological reports of TEN cases in Japan.	The relevant information is presented in the Methods section and Figure 1.
42			2	• For each variable of interest, the authors should clearly describe the sources of data and the methods of measurement or assessment. This includes the procedures used for serological testing and immunoblot analysis.	The Methods section has been revised accordingly.
43			2	The clinical characteristics of the study population are insufficiently described. Additional information should be provided regarding:<br>• age,<br>• sex,<br>• other relevant comorbidities that may influence serological responses.	The additional information have been included in Table 1.
44			2	• The treatment regimens administered to the patients are not described, and information on treatment adherence is also lacking. Since treatment may directly affect serological kinetics, this information is essential for interpretation of the results.	The additional information have been included in the Methods section and Table 1.
45			2	• The follow-up intervals were highly variable (31–515 days). In several cases, the minimum follow-up period of six months typically required to evaluate meaningful serological decline was not completed. The authors should discuss how this variability may affect interpretation of antibody kinetics.	The time interval have been limited to: the initial visit (day 0) and post-treatment follow-up (30–189 days after completion of treatment).

46			2	• The molecular diagnostic methods to Treponemal identification require further clarification. In particular, the authors should specify from which clinical sample the DNA was extracted (e.g., serum, lesion swab, or tissue sample).	The additional information have been included in the Methods section and Table 1.
47			2	• The manuscript would benefit from a clearly articulated hypothesis. For example:<br>"We hypothesized that the kinetics of TP and RPR antibody titers following treatment in TEN infection are comparable to those observed in TPA infection."<br>Explicitly stating the hypothesis would strengthen the conceptual framework and analytical approach of the study.	The manuscript has been revised in accordance with your comment.
48			2	• The selection of the comparator group (TPA cases) is not clearly described. The authors should clarify:<br>• how the five TPA cases were selected,<br>• whether they were consecutive cases, and<br>• whether any matching criteria (e.g., age, stage of disease, treatment regimen) were applied.	The additional information have been included in the Methods section and Figure 1.
49	Conclusions		Journal	・Are the conclusions supported by the data presented?<br>・Are the limitations of analysis clearly described?<br>・Are the conclusions supported by the data presented?<br>・Do the authors discuss how these data can be helpful to advance our understanding of the topic under study?<br>・Is public health relevance addressed?	The manuscript has been revised in accordance with your comment.
50			2	• In the limitations section, the authors should explicitly acknowledge the small number of patients included in the study, as well as the lack of standardized treatment and follow-up protocols.	The manuscript has been revised in accordance with your comment.
51			2	• Additional limitations should also be discussed, including:<br>o the restriction to a single geographic region, which may limit generalizability, and<br>o the heterogeneity in the clinical stages of infection among the included cases.	The Discussion section has been revised in accordance with your comment.
52			2	• The authors highlight the potential utility of the serological tests, stating that:<br>"Serological testing demonstrated that both TEN- and TPA-infected patients showed similar patterns of treponemal (TP) and non-treponemal (RPR) antibody responses."<br>• However, given the small sample size and the exploratory nature of the study, the conclusions should be moderated to avoid overinterpretation of the findings.<br>• Suggested revision of the conclusion:<br>"Our findings suggest that serological responses in TEN infection may resemble those observed in TPA infection."	The manuscript has been revised in accordance with your comment.
53			2	• The manuscript would also benefit from a stronger discussion of the broader clinical and public health implications, particularly regarding:<br>o the emergence of sexually transmitted TEN infections,<br>o the diagnostic implications in non-endemic settings, and<br>o the potential for misclassification of TEN infections as syphilis.	The Introduction and Discussion has been revised in accordance with your comment.
54			2	• These issues are particularly relevant for epidemiological surveillance and STI control programs, where accurate differentiation between TEN and TPA infections may have implications for case reporting and public health monitoring.	The Introduction and Discussion has been revised in accordance with your comment.
55	Editorial and Data Presentation Modifications?		Journal	Use this section for editorial suggestions as well as relatively minor modifications of existing data that would enhance clarity. If the only modifications needed are minor and/or editorial, you may wish to recommend "Minor Revision" or "Accept".	-
56			2	• The results are generally presented clearly, but some figures and tables could be improved to enhance readability. For example:<br>o Figure 1 is dense and difficult to interpret due to multiple panels with differing axes.<br>o Summary statistics (e.g., medians and interquartile ranges) could complement the individual-level trajectories.<br>o Some figures could be moved to supplementary material to streamline the main text.	The manuscript has been revised in accordance with your comment.
