# Supplementary material for: Evaluation of treatment-related serological changes in Treponema pallidum subsp. endemicum infection using latex-based immunoassays, with comparison to Treponema pallidum subsp. pallidum
Source: PLoS Negl Trop Dis. 2026 Oct 15;20(10):e0014756. doi: 10.1371/journal.pntd.0014756 (PMC13671511; doi:10.1371/journal.pntd.0014756)
Supplement: Supplementary file 2 [file pntd.0014756.s006.pdf]

No.	Section1	Reviewer/ Journal	Comment/Question	Answer	Changes in the revised manuscript
1	General	Journal	1)Thank you for stating "All relevant data are within the paper and its Supporting Information files. The accession numbers for tp0548 and tp0856 sequences from bejel and syphilis cases are provided in Table 1. The corresponding nucleotide sequence data have been deposited in the DNA Data Bank of Japan (DDBJ) under accession numbers LC383799, LC383801, LC430602–LC430605, LC430607–LC430608, LC589188–LC589190, and LC931913–LC931924." However, we were unable to access the data using the information provided. Please review and update the Data Availability Statement to ensure that the underlying data are publicly accessible and that all accession numbers, links, or repository details are correct and functional.	Thank you for pointing this out. The nucleotide sequence data have now been made publicly accessible in the DNA Data Bank of Japan (DDBJ). We have confirmed that all accession numbers listed in the Data Availability Statement are now accessible.	
2	Methods	2	The manuscript's methodology is appropriate for investigating a very rare infection. The most important revisions are not to increase the sample size—which is likely infeasible—but to present the work explicitly as an exploratory study, specify that the statistical analisys is descriptive.	We have clarified that this study should be regarded as an exploratory study and that the findings are presented descriptively because of the limited number of genetically confirmed bejel cases.	Page 10, Lines 140–142: We added a statement clarifying that, because of the rarity of bejel and the limited number of eligible cases, this study should be regarded as exploratory and that the results are presented descriptively.
3	Results	2	The Results section is scientifically coherent and substantially improved in the revised manuscript.		
4	Conclusions	2	The Discussion and Conclusions are scientifically sound and highlight the novelty and clinical importance of the study. However, the conclusions should be tempered to reflect the exploratory nature of the data.	Thank you for this valuable comment. In response to the reviewer's suggestion, we have revised the Discussion and Conclusion to more clearly reflect the exploratory nature of this study and to avoid overinterpretation of the findings. Specifically, we revised the Conclusion to state that the findings are derived from an exploratory study and that the observed antibody dynamics in bejel only appear similar to those in syphilis.<br>We also modified the Discussion to use more cautious language when describing the serological responses in bejel and revised the interpretation to avoid implying equivalence between bejel and syphilis. Finally, we expanded the limitations by emphasizing that the findings should be interpreted cautiously because of the exploratory nature of the data and require confirmation in larger studies.	Page 22, Lines 326–327, Page 23, Lines 355–357, Page 24, Line 366–368, Pages 24, Lines 371–373: The Discussion and Conclusion were revised to temper the interpretation of the findings by emphasizing the exploratory nature of the study, using more cautious language throughout, and expanding the limitations to recommend confirmation in larger studies.
5	Editorial and Data Presentation Modifications?	2	NA		
6	Editorial and Data Presentation Modifications?	3	There are still a few minor issues that should be addressed:<br><br>1- Methodology (lines 134–146): This section appears to be somewhat redundant and could be streamlined for clarity and conciseness.<br><br>2- Results (Table 2): It would be more informative to report the comparisons of TP-LA vs. TPPA and RPR-LA vs. RPR for each time point separately.<br><br>3- Figures: The graphical quality of the figures could still be improved. I suggest using R or other specialized data visualization tools instead of MS Office to enhance presentation quality.	1- We appreciate the reviewer's helpful suggestion. To improve clarity and conciseness, we revised the "Sample selection" section. Specifically, we summarized the patient selection process in the main text and referred readers to Figure 1 for the detailed case-selection flow, thereby avoiding redundancy with the figure legend.<br><br>2- Thank you for this helpful suggestion. We agree that separate comparisons for the pre-treatment and post-treatment samples provide additional information. Accordingly, we performed separate regression analyses for the pre-treatment and post-treatment subgroups and present the results in Supplementary Table S1. Because these data are supplementary to the main findings and would substantially increase the size of Table 2, we have presented them as Supplementary Table S1, while retaining the overall comparison in the main manuscript.<br>The Results section has also been revised to refer readers to Supplementary Table S1 for these additional analyses. The original supplementary table containing the individual serological and immunoblot data underlying Figures 3 and 4 has been renumbered as Supplementary Table S2, and the corresponding citations in the manuscript have been updated accordingly. In addition, during preparation of Supplementary Table S1, we identified minor errors in the regression equations and coefficients of determination in Table 2, which have now been corrected. We also added the N column and revised the coefficient of determination column heading to R^2 for consistency with Supplementary Table S1.<br><br>3- Thank you for this helpful suggestion. All figures have been regenerated as high-resolution TIFF images (600 dpi) suitable for publication. We believe that the graphical quality and readability of the figures have been substantially improved.	1-<br>Pages 9–10, Lines 133–142: The "Sample selection" section was streamlined by summarizing the patient selection process and referring readers to Figure 1 for the detailed flow of case selection, thereby avoiding redundancy with the figure legend.<br><br>2-<br>Pages 17–18, Lines 260–261, Page 31, Lines 507–514: Supplementary Table S1 was added to present separate regression analyses for the pre-treatment and post-treatment subgroups. The Results section was revised accordingly, and the original supplementary table was renumbered as Supplementary Table S2.<br>Page 17, Lines 253–254: Minor errors in the regression equations and coefficients of determination were corrected. In addition, the N column was added, and the coefficient of determination column heading was revised to R^2 for consistency with Supplementary Table S1.<br><br>3-<br>All figures were regenerated as high-resolution TIFF images (600 dpi) to improve graphical quality and readability.
7	Summary and General Comments	1	I am satisfied with the changes made.		
8	Summary and General Comments	2	This revised manuscript addresses an important and understudied topic by evaluating treatment-related serological responses in genetically confirmed Treponema pallidum subsp. endemicum infection. The study is novel and clinically relevant, particularly given the emergence of sexually transmitted bejel outside endemic regions. The revised version has improved substantially, with clearer objectives, better organization, and more transparent methodology. However, the small sample size limits statistical power, and the analyses should be consistently presented as exploratory. The conclusions should be tempered to avoid implying equivalence between TEN and TPA. No additional experiments are required; the requested revisions primarily involve improving statistical reporting, interpretation, presentation of the results, and discussion of study limitations. Overall, after these revisions, the manuscript would make a valuable contribution to the literature on emerging endemic treponematoses.	We sincerely thank the reviewer for the positive overall assessment of our manuscript and for the constructive suggestions.<br>In accordance with the reviewer's comments, we have revised the manuscript to consistently present the study as exploratory, tempered the interpretation of the findings and conclusions, expanded the discussion of the study limitations, and clarified that the analyses are descriptive in nature.<br>We also improved the overall presentation and clarity of the manuscript, including streamlining the methodology. No additional experiments were performed, consistent with the reviewer's recommendation.	
9	Summary and General Comments	3	The manuscript has been improved, and the authors have addressed most of my comments. I can recommend this manuscript to be considered for publication in PLOS Neglected Tropical Diseases.		
