# Supplementary material for: Evaluation of treatment-related serological changes in Treponema pallidum subsp. endemicum infection using latex-based immunoassays, with comparison to Treponema pallidum subsp. pallidum
Source: PLoS Negl Trop Dis. 2026 Oct 15;20(10):e0014756. doi: 10.1371/journal.pntd.0014756 (PMC13671511; doi:10.1371/journal.pntd.0014756)
Supplement: Peer review history

# Peer review: Decision Letter 0

**DOI:** 10.1371/journal.pntd.0014756.r001

**Authors:** Elsio A Wunder Jr, Kelly L. Hawley

17 Mar 2026

Serological characteristics and treatment-related changes in Treponema pallidum subsp. endemicum infection: Comparison with Treponema pallidum subsp. Pallidum.

PLOS Neglected Tropical Diseases

Dear Dr. Deno,

Thank you for submitting your manuscript to PLOS Neglected Tropical Diseases. After careful consideration, we feel that it has merit but does not fully meet PLOS Neglected Tropical Diseases's publication criteria as it currently stands. Therefore, we invite you to submit a revised version of the manuscript that addresses the points raised during the review process.

Please submit your revised manuscript within by May 16 2026 11:59PM. If you will need more time than this to complete your revisions, please reply to this message or contact the journal office at plosntds@plos.org. When you're ready to submit your revision, log on to https://www.editorialmanager.com/pntd/ and select the 'Submissions Needing Revision' folder to locate your manuscript file.

Please include the following items when submitting your revised manuscript:

* A letter that responds to each point raised by the editor and reviewer(s). You should upload this letter as a separate file labeled 'Response to Reviewers'. This file does not need to include responses to any formatting updates and technical items listed in the 'Journal Requirements' section below.

* A marked-up copy of your manuscript that highlights changes made to the original version. You should upload this as a separate file labeled 'Revised Manuscript with Track Changes'.

* An unmarked version of your revised paper without tracked changes. You should upload this as a separate file labeled 'Manuscript'.

If you would like to make changes to your financial disclosure, competing interests statement, or data availability statement, please make these updates within the submission form at the time of resubmission. Guidelines for resubmitting your figure files are available below the reviewer comments at the end of this letter.

We look forward to receiving your revised manuscript.

Kind regards,

Kelly L. Hawley

Academic Editor

PLOS Neglected Tropical Diseases

Elsio Wunder Jr

Section Editor

PLOS Neglected Tropical Diseases

Shaden Kamhawi

co-Editor-in-Chief

PLOS Neglected Tropical Diseases

orcid.org/0000-0003-4304-636XX

Paul Brindley

co-Editor-in-Chief

PLOS Neglected Tropical Diseases

orcid.org/0000-0003-1765-0002

**Additional Editor Comments (if provided):**

**Journal Requirements:**

If the reviewer comments include a recommendation to cite specific previously published works, please review and evaluate these publications to determine whether they are relevant and should be cited. There is no requirement to cite these works unless the editor has indicated otherwise.

[Note: HTML markup is below. Please do not edit.]

**Reviewers' Comments:**

Reviewer's Responses to Questions

**Results**

-Does the analysis presented match the analysis plan?

-Are the results clearly and completely presented?

-Are the figures (Tables, Images) of sufficient quality for clarity?

Reviewer #2: • The results are presented clearly and in sufficient detail, and the tables and figures provide useful information to understand the serological dynamics observed in the study population.

• Clarifications are needed regarding Table 1:

o TEN Case 1:

Why is there a second visit labeled “TEN infection, before treatment”?

Please clarify whether treatment was not initiated immediately after the first visit or whether there was a delay between diagnosis and treatment initiation.

o TEN Case 2:

Why is the first visit labeled “TEN infection, in treatment” and the second visit still described as “TEN infection, after treatment” rather than “Resolved”?

Clarification of the clinical status terminology used in the table would improve interpretability.

• Tables and figures:

o Tables 1 and 2 are detailed and provide essential information on the clinical findings and antibody profiles of the included cases.

o Table 2 and Figures 2, 3, and 4 appear to present partially overlapping or complementary information.

The authors may consider moving some of these figures to the supplementary material to improve the readability and flow of the main manuscript.

• Figure 1:

o Figure 1, which presents the changes in antibody titers over time, is currently very dense and difficult to interpret visually.

o Suggestion for improvement:

� Standardize the axis scales across panels, and

� Consider grouping the graphs more clearly to facilitate immediate visual comparison between TPA and TEN cases or primary vs secondary.

• Statistical analysis:

o The manuscript reports that a two-tailed Welch’s t-test was used to compare the rates of change in antibody titers.

o Given the very small sample size (n < 30), the authors should clarify:

� whether the normality of the data distribution was assessed, and

� whether a non-parametric test (e.g., Mann–Whitney U test) might provide a more robust comparison under these conditions.

Reviewer #3: 1- The authors did not provide any phylogenetic analysis; without a reference phylogeny showing how their isolates cluster relative to established TPA and TEN lineages, their proposed subspecies assignments remain unsubstantiated.

2-In general, the quality of the figures needs to be improved; all axes should be clearly and consistently labeled to ensure the data can be interpreted accurately.

**********

**Summary and General Comments**

Use this section to provide overall comments, discuss strengths/weaknesses of the study, novelty, significance, general execution and scholarship. You may also include additional comments for the author, including concerns about dual publication, research ethics, or publication ethics. If requesting major revision, please articulate the new experiments that are needed.

Reviewer #1: This is a straightforward paper. The results are I think unsurprising given the existing literature but there are few recent papers on endemicum espcially in high income settings.

The introduction reads as if we dont really know anything about the performance of serology in the endemic treponematoses but of course the performance of these tests in yaws including both treponemal and non-treponemal tests is extremely well described including for monitoring response to treatment - there are many papers on this. I think it would be helpful to incorporate this because the a priori position I think would be to anticipate that serology performs identically between TPE/TPA/TPP. Similarly the discussion totally omits any discussion of what we already know about antibody responses in non-venereal trepoentmatoses and needs to be re-written to incorporate that knowledge.

Please try and use person centred language and avoid describing people as 'TEN infected' etc.

Please describe in the methods the timing of sample collection - i.e was followup at standard time points or at the discretion of the treating clinicians? The statistical comparisons between groups arent really described in the methods. If you are going to present a comparison please make sure that is specified in the methods.

I think most readers will find the way the RPR titres are described confusing - did you not get standard titres i.e 1:1, 1:2, 1:4 etc? I think clinical readers will want to understand the fall in terms of dilutions over time not units per day.

In general I think the results are too long for what is essentially a small comparison of 2 groups of about 5 patients each. They could be greatly simplified to describe baseline and endline titres with 1-2 tables or figures maximum.

Reviewer #2: • This manuscript examines the serological characteristics and treatment-related changes in Treponema pallidum subsp. endemicum (TEN) infection and compares them with those observed in Treponema pallidum subsp. pallidum (TPA) infection. The topic is timely and relevant, particularly in light of recent reports of sexually transmitted TEN infections outside traditionally endemic regions.

• The study addresses an important diagnostic and epidemiological question: whether conventional serological assays developed for syphilis can be applied to TEN infection and whether antibody kinetics after treatment behave similarly in both infections. This issue has practical implications for clinical diagnosis, laboratory interpretation of serology, and surveillance systems, particularly in non-endemic settings where TEN infections may be misclassified as syphilis.

• The manuscript has several notable strengths:

o The study includes genetically confirmed TEN cases, which are rarely reported in the literature.

o The authors combine molecular identification with serological and immunoblot analyses, providing a multidimensional characterization of the infections.

o The comparison between TEN and TPA infections is conceptually relevant, especially in the context of emerging sexually transmitted TEN cases.

o The manuscript provides detailed individual-level serological data, which may contribute valuable preliminary information to a limited evidence base.

• Despite these strengths, the study also has important methodological limitations that should be addressed before publication.

1. Major methodological limitations

• The sample size is extremely small (n = 11; 6 TEN and 5 TPA cases). While the rarity of sexually transmitted TEN infections is acknowledged, this limited sample size substantially restricts the statistical power and generalizability of the findings.

• The manuscript currently applies inferential statistical tests (Welch’s t-test) despite the very small sample size and the exploratory nature of the dataset. The authors should clarify whether the study should be considered exploratory/descriptive rather than hypothesis-testing.

• The study design and patient selection process are not sufficiently described. The manuscript should clarify:

o the recruitment period,

o the selection criteria for inclusion,

o whether the TEN cases represent all identified cases during the study period, and

o how the TPA comparator cases were selected.

• The clinical characterization of the patients is limited. Additional information regarding age, sex, HIV status, comorbidities, and stage of infection would improve interpretation of the serological findings.

• The treatment regimens administered and treatment adherence are not described, yet treatment could directly influence serological kinetics.

• The follow-up intervals were highly variable (31–515 days), and in some cases the recommended follow-up period for evaluating serological decline after treatment was not completed. This variability should be acknowledged as a limitation.

• The laboratory methods require clarification, particularly regarding:

o the clinical sample type used for DNA extraction, and

o the criteria used to classify cases as TEN versus TPA when sequence data were incomplete.

2. Interpretation of findings

• The authors conclude that serological assays developed for syphilis may be applicable to TEN infection. While the data suggest that similar antibody patterns may occur, the current evidence should be interpreted cautiously due to the small sample size and exploratory design.

• The conclusions would benefit from more cautious wording. For example:

“Our findings suggest that serological responses in TEN infection may resemble those observed in TPA infection.”

• The manuscript would also benefit from a more detailed discussion of the broader clinical and public health implications, including:

o the emergence of sexually transmitted TEN infections,

o the diagnostic challenges in non-endemic settings, and

o the potential misclassification of TEN infection as syphilis in surveillance systems.

3. Data presentation

• The results are generally presented clearly, but some figures and tables could be improved to enhance readability. For example:

o Figure 1 is dense and difficult to interpret due to multiple panels with differing axes.

o Summary statistics (e.g., medians and interquartile ranges) could complement the individual-level trajectories.

o Some figures could be moved to supplementary material to streamline the main text.

Recommendation

• Overall, this manuscript addresses a relevant and underexplored topic and provides preliminary clinical and serological observations on TEN infections in a non-endemic setting. However, the methodological description, interpretation of findings, and discussion of limitations require substantial clarification.

• For these reasons, minor revision is recommended.

• The revision should focus on:

o clarifying the study design and patient selection,

o improving the description of the study population and treatments,

o moderating the interpretation of the findings,

o strengthening the discussion of limitations and public health relevance, and

o improving the presentation of the results and figures.

Reviewer #3: This study addresses an interesting and clinically relevant topic. Understanding how treponemal versus nontreponemal serology behaves across T. pallidum subspecies and how titers evolve post-treatment would substantially inform diagnostics and patient management. However, Deno et al. do not provide the statistical rigor required to support their central claim: "The serological profiles and post-treatment changes in TEN-infected patients were similar to those observed in TPA-infected patients."

Major concerns:

1- Severe underpowering precludes equivalence claims

With n=6 TEN vs n=5 TPA, the study can only detect extremely large between-group differences. A non-significant Welch t-test (p > 0.05) may reflect this low power rather than evidence of equivalence. This could be the classic Type II error risk and non-significant results cannot substantiate "similarity." If the authors intend to claim practical equivalence (i.e., differences are clinically negligible), they could use methods like equivalence testing (e.g., TOST) with a priori justified equivalence bounds (SESOI) and demonstrate that the 90% CI for the group difference falls entirely within those bounds.

The TP/day and RPR/day analyses are particularly fragile given extreme outliers (e.g. -361.8, -218.6 T.U./day), marked skewness, and high variability (SDs around 100-160 for T.U./day). These properties violate parametric t-test assumptions and render confidence intervals uninformative. The non-significant results should be framed as inconclusive. Authors should:

a- Reframe as exploratory analyses

b- Report effect sizes (Cohen's d) with 95% CIs

c- Present medians/IQRs alongside means

d- Consider non-parametric alternatives (Mann-Whitney on change scores)

2- tp0548/tp0856 sequencing lacks validation and documentation:

Phylogeny based on tp0548 and tp0856 is not an established method for TPE/TEN discrimination. The authors fail to justify bypassing the validated 7-locus MLST scheme (Pal-Diaz et al., 2025) or gold-standard whole-genome sequencing. Methodological transparency is also inadequate and no details are provided on sequencing platform (Sanger vs. NGS), bioinformatics pipeline (reference genome, variant calling), or phylogenetic context.

**********

PLOS authors have the option to publish the peer review history of their article (what does this mean?). If published, this will include your full peer review and any attached files.

If you choose “no”, your identity will remain anonymous but your review may still be made public.

**Do you want your identity to be public for this peer review?**  For information about this choice, including consent withdrawal, please see our Privacy Policy.

Reviewer #1: No

Reviewer #2: No

Reviewer #3: No

**********

**Key Review Criteria Required for Acceptance?**

As you describe the new analyses required for acceptance, please consider the following:

**Methods**

-Are the objectives of the study clearly articulated with a clear testable hypothesis stated?

-Is the study design appropriate to address the stated objectives?

-Is the population clearly described and appropriate for the hypothesis being tested?

-Is the sample size sufficient to ensure adequate power to address the hypothesis being tested?

-Were correct statistical analysis used to support conclusions?

-Are there concerns about ethical or regulatory requirements being met?

Reviewer #2: • The study is described as having an observational, prospective, and analytical design, which allows comparison of the serological behavior between patients infected with Treponema pallidum subsp. pallidum (TPA) and Treponema pallidum subsp. endemicum (TEN). However, the manuscript should explicitly state the study design early in the Methods section (e.g., prospective observational cohort or prospective case series with comparative analysis).

• The study setting is insufficiently described. The authors should clearly specify:

• the clinical setting at the institutions involved,

• the time period of recruitment,

• It remains unclear whether the samples were originally collected for routine clinical care or for research purposes. Clarification is required to understand the context of sample collection and potential selection bias.

• Eligibility criteria and participant selection should be described in greater detail. Specifically, the manuscript should report:

• the inclusion and exclusion criteria,

• the sources of participant recruitment,

• the methods used for participant selection, and

• the procedures used for follow-up assessments.

• The study population is extremely small (6 TEN cases and 5 TPA cases). While it is acknowledged that sexually transmitted TEN in non-endemic settings is rare, the authors should address whether this sample size is sufficient to ensure adequate statistical power to detect meaningful differences between groups.

• In its current form, the manuscript does not include a sample size calculation or power estimation. The authors should either:

• provide a power calculation, or

• explicitly acknowledge that the study should be interpreted as exploratory or descriptive.

• The representativeness of the included cases should be justified. In particular, the authors should clarify whether these 11 cases represent:

• all identified cases during the study period, or

• a selected subset.

This clarification is important in the context of the recent epidemiological reports of TEN cases in Japan.

• For each variable of interest, the authors should clearly describe the sources of data and the methods of measurement or assessment. This includes the procedures used for serological testing and immunoblot analysis.

The clinical characteristics of the study population are insufficiently described. Additional information should be provided regarding:

• age,

• sex,

• other relevant comorbidities that may influence serological responses.

• The treatment regimens administered to the patients are not described, and information on treatment adherence is also lacking. Since treatment may directly affect serological kinetics, this information is essential for interpretation of the results.

• The follow-up intervals were highly variable (31–515 days). In several cases, the minimum follow-up period of six months typically required to evaluate meaningful serological decline was not completed. The authors should discuss how this variability may affect interpretation of antibody kinetics.

• The molecular diagnostic methods to Treponemal identification require further clarification. In particular, the authors should specify from which clinical sample the DNA was extracted (e.g., serum, lesion swab, or tissue sample).

• The manuscript would benefit from a clearly articulated hypothesis. For example:

“We hypothesized that the kinetics of TP and RPR antibody titers following treatment in TEN infection are comparable to those observed in TPA infection.”

Explicitly stating the hypothesis would strengthen the conceptual framework and analytical approach of the study.

• The selection of the comparator group (TPA cases) is not clearly described. The authors should clarify:

• how the five TPA cases were selected,

• whether they were consecutive cases, and

• whether any matching criteria (e.g., age, stage of disease, treatment regimen) were applied.

**********

**Conclusions**

-Are the conclusions supported by the data presented?

-Are the limitations of analysis clearly described?

-Do the authors discuss how these data can be helpful to advance our understanding of the topic under study?

-Is public health relevance addressed?

Reviewer #2: • In the limitations section, the authors should explicitly acknowledge the small number of patients included in the study, as well as the lack of standardized treatment and follow-up protocols.

• Additional limitations should also be discussed, including:

o the restriction to a single geographic region, which may limit generalizability, and

o the heterogeneity in the clinical stages of infection among the included cases.

• The authors highlight the potential utility of the serological tests, stating that:

“Serological testing demonstrated that both TEN- and TPA-infected patients showed similar patterns of treponemal (TP) and non-treponemal (RPR) antibody responses.”

• However, given the small sample size and the exploratory nature of the study, the conclusions should be moderated to avoid overinterpretation of the findings.

• Suggested revision of the conclusion:

“Our findings suggest that serological responses in TEN infection may resemble those observed in TPA infection.”

• The manuscript would also benefit from a stronger discussion of the broader clinical and public health implications, particularly regarding:

o the emergence of sexually transmitted TEN infections,

o the diagnostic implications in non-endemic settings, and

o the potential for misclassification of TEN infections as syphilis.

• These issues are particularly relevant for epidemiological surveillance and STI control programs, where accurate differentiation between TEN and TPA infections may have implications for case reporting and public health monitoring.

**********

**Editorial and Data Presentation Modifications?**

Use this section for editorial suggestions as well as relatively minor modifications of existing data that would enhance clarity. If the only modifications needed are minor and/or editorial, you may wish to recommend “Minor Revision” or “Accept”.

Reviewer #2: • The results are generally presented clearly, but some figures and tables could be improved to enhance readability. For example:

o Figure 1 is dense and difficult to interpret due to multiple panels with differing axes.

o Summary statistics (e.g., medians and interquartile ranges) could complement the individual-level trajectories.

o Some figures could be moved to supplementary material to streamline the main text.

[NOTE: If reviewer comments were submitted as an attachment file, they will be attached to this email and accessible via the submission site. Please log into your account, locate the manuscript record, and check for the action link "View Attachments". If this link does not appear, there are no attachment files.]

**Figure resubmission:**

**Reproducibility:**

To enhance the reproducibility of your results, we recommend that authors of applicable studies deposit laboratory protocols in protocols.io, where a protocol can be assigned its own identifier (DOI) such that it can be cited independently in the future. Additionally, PLOS ONE offers an option to publish peer-reviewed clinical study protocols. Read more information on sharing protocols at https://plos.org/protocols?utm_medium=editorial-email&utm_source=authorletters&utm_campaign=protocols

# Author comment: Author response to Decision Letter 1

**DOI:** 10.1371/journal.pntd.0014756.r002

24 May 2026

Attachment Submitted filename: Response to reviewers.pdf

# Peer review: Decision Letter 1

**DOI:** 10.1371/journal.pntd.0014756.r003

**Authors:** Elsio A Wunder Jr, Kelly L. Hawley

14 Jul 2026

Response to Reviewers Revised Manuscript with Track Changes Manuscript

Shaden Kamhawi

co-Editor-in-Chief

PLOS Neglected Tropical Diseases

orcid.org/0000-0003-4304-636XX

Paul Brindley

co-Editor-in-Chief

PLOS Neglected Tropical Diseases

orcid.org/0000-0003-1765-0002

**Additional Editor Comments (if provided):** **Journal Requirements:**

1)Thank you for stating "All relevant data are within the paper and its Supporting Information files. The accession numbers for tp0548 and tp0856 sequences from bejel and syphilis cases are provided in Table 1. The corresponding nucleotide sequence data have been deposited in the DNA Data Bank of Japan (DDBJ) under accession numbers LC383799, LC383801, LC430602–LC430605, LC430607–LC430608, LC589188–LC589190, and LC931913–LC931924." However, we were unable to access the data using the information provided. Please review and update the Data Availability Statement to ensure that the underlying data are publicly accessible and that all accession numbers, links, or repository details are correct and functional.

**Reviewers' comments:**

**Key Review Criteria Required for Acceptance?**

As you describe the new analyses required for acceptance, please consider the following:

**Methods**

-Are the objectives of the study clearly articulated with a clear testable hypothesis stated?

-Is the study design appropriate to address the stated objectives?

-Is the population clearly described and appropriate for the hypothesis being tested?

-Is the sample size sufficient to ensure adequate power to address the hypothesis being tested?

-Were correct statistical analysis used to support conclusions?

-Are there concerns about ethical or regulatory requirements being met?

Reviewer #1: (No Response)

Reviewer #2: The manuscript's methodology is appropriate for investigating a very rare infection. The most important revisions are not to increase the sample size—which is likely infeasible—but to present the work explicitly as an exploratory study, specify that the statistical analisys is descriptive.

Reviewer #3: (No Response)

**********

**Results**

-Does the analysis presented match the analysis plan?

-Are the results clearly and completely presented?

-Are the figures (Tables, Images) of sufficient quality for clarity?

Reviewer #1: (No Response)

Reviewer #2: The Results section is scientifically coherent and substantially improved in the revised manuscript.

Reviewer #3: (No Response)

**********

**Conclusions**

-Are the conclusions supported by the data presented?

-Are the limitations of analysis clearly described?

-Do the authors discuss how these data can be helpful to advance our understanding of the topic under study?

-Is public health relevance addressed?

Reviewer #1: (No Response)

Reviewer #2: The Discussion and Conclusions are scientifically sound and highlight the novelty and clinical importance of the study. However, the conclusions should be tempered to reflect the exploratory nature of the data.

Reviewer #3: (No Response)

**********

**Editorial and Data Presentation Modifications?**

Use this section for editorial suggestions as well as relatively minor modifications of existing data that would enhance clarity. If the only modifications needed are minor and/or editorial, you may wish to recommend “Minor Revision” or “Accept”.

Reviewer #1: (No Response)

Reviewer #2: NA

Reviewer #3: There are still a few minor issues that should be addressed:

1- Methodology (lines 134–146): This section appears to be somewhat redundant and could be streamlined for clarity and conciseness.

2- Results (Table 2): It would be more informative to report the comparisons of TP-LA vs. TPPA and RPR-LA vs. RPR for each time point separately.

3- Figures: The graphical quality of the figures could still be improved. I suggest using R or other specialized data visualization tools instead of MS Office to enhance presentation quality.

**********

**Summary and General Comments**

Use this section to provide overall comments, discuss strengths/weaknesses of the study, novelty, significance, general execution and scholarship. You may also include additional comments for the author, including concerns about dual publication, research ethics, or publication ethics. If requesting major revision, please articulate the new experiments that are needed.

Reviewer #1: I am satisfied with the changes made.

Reviewer #2: This revised manuscript addresses an important and understudied topic by evaluating treatment-related serological responses in genetically confirmed Treponema pallidum subsp. endemicum infection. The study is novel and clinically relevant, particularly given the emergence of sexually transmitted bejel outside endemic regions. The revised version has improved substantially, with clearer objectives, better organization, and more transparent methodology. However, the small sample size limits statistical power, and the analyses should be consistently presented as exploratory. The conclusions should be tempered to avoid implying equivalence between TEN and TPA. No additional experiments are required; the requested revisions primarily involve improving statistical reporting, interpretation, presentation of the results, and discussion of study limitations. Overall, after these revisions, the manuscript would make a valuable contribution to the literature on emerging endemic treponematoses.

Reviewer #3: The manuscript has been improved, and the authors have addressed most of my comments. I can recommend this manuscript to be considered for publication in PLOS Neglected Tropical Diseases.

**********

PLOS authors have the option to publish the peer review history of their article (what does this mean?). If published, this will include your full peer review and any attached files.

If you choose “no”, your identity will remain anonymous but your review may still be made public.

**Do you want your identity to be public for this peer review?**  For information about this choice, including consent withdrawal, please see our Privacy Policy.

Reviewer #1: No

Reviewer #2: No

Reviewer #3: No

**Figure resubmission:**

**Reproducibility:**

To enhance the reproducibility of your results, we recommend that authors of applicable studies deposit laboratory protocols in protocols.io, where a protocol can be assigned its own identifier (DOI) such that it can be cited independently in the future. Additionally, PLOS ONE offers an option to publish peer-reviewed clinical study protocols. Read more information on sharing protocols at https://plos.org/protocols?utm_medium=editorial-email&utm_source=authorletters&utm_campaign=protocols

# Author comment: Author response to Decision Letter 2

**DOI:** 10.1371/journal.pntd.0014756.r004

17 Aug 2026

Attachment Submitted filename: Response to reviewersR2.pdf

# Editor report: Decision Letter 2

**DOI:** 10.1371/journal.pntd.0014756.r005

**Authors:** Elsio A Wunder Jr, Kelly L. Hawley

15 Sep 2026

Dear Mr. Deno,

We are pleased to inform you that your manuscript 'Evaluation of treatment-related serological changes in Treponema pallidum subsp. endemicum infection using latex-based immunoassays, with comparison to Treponema pallidum subsp. pallidum' has been provisionally accepted for publication in PLOS Neglected Tropical Diseases.

Before your manuscript can be formally accepted you will need to complete some formatting changes, which you will receive in a follow up email. A member of our team will be in touch with a set of requests.

Please note that your manuscript will not be scheduled for publication until you have made the required changes, so a swift response is appreciated.

IMPORTANT: The editorial review process is now complete. PLOS will only permit corrections to spelling, formatting or significant scientific errors from this point onwards. Requests for major changes, or any which affect the scientific understanding of your work, will cause delays to the publication date of your manuscript.

Should you, your institution's press office or the journal office choose to press release your paper, you will automatically be opted out of early publication. We ask that you notify us now if you or your institution is planning to press release the article. All press must be co-ordinated with PLOS.

Thank you again for supporting Open Access publishing; we are looking forward to publishing your work in PLOS Neglected Tropical Diseases.

Best regards,

Kelly L. Hawley

Academic Editor

PLOS Neglected Tropical Diseases

Elsio Wunder Jr

Section Editor

PLOS Neglected Tropical Diseases

Shaden Kamhawi

co-Editor-in-Chief

PLOS Neglected Tropical Diseases

orcid.org/0000-0003-4304-636XX

Paul Brindley

co-Editor-in-Chief

PLOS Neglected Tropical Diseases

orcid.org/0000-0003-1765-0002

***********************************************************

p.p1 {margin: 0.0px 0.0px 0.0px 0.0px; line-height: 16.0px; font: 14.0px Arial; color: #323333; -webkit-text-stroke: #323333}span.s1 {font-kerning: none

# Editor report: Acceptance letter

**DOI:** 10.1371/journal.pntd.0014756.r006

**Authors:** Elsio A Wunder Jr, Kelly L. Hawley

Dear Mr. Deno,

We are delighted to inform you that your manuscript, "Evaluation of treatment-related serological changes in Treponema pallidum subsp. endemicum infection using latex-based immunoassays, with comparison to Treponema pallidum subsp. pallidum," has been formally accepted for publication in PLOS Neglected Tropical Diseases.

We have now passed your article onto the PLOS Production Department who will complete the rest of the publication process. All authors will receive a confirmation email upon publication.

The corresponding author will soon be receiving a typeset proof for review, to ensure errors have not been introduced during production. Please review the PDF proof of your manuscript carefully, as this is the last chance to correct any scientific or type-setting errors. Please note that major changes, or those which affect the scientific understanding of the work, will likely cause delays to the publication date of your manuscript. Note: Proofs for Front Matter articles (Editorial, Viewpoint, Symposium, Review, etc...) are generated on a different schedule and may not be made available as quickly.

Soon after your final files are uploaded, the early version of your manuscript will be published online unless you opted out of this process. The date of the early version will be your article's publication date. The final article will be published to the same URL, and all versions of the paper will be accessible to readers.

For Research Articles, you will receive an invoice from PLOS for your publication fee after your manuscript has reached the completed accept phase. If you receive an email requesting payment before acceptance or for any other service, this may be a phishing scheme. Learn how to identify phishing emails and protect your accounts at https://explore.plos.org/phishing.

Thank you again for supporting open-access publishing; we are looking forward to publishing your work in PLOS Neglected Tropical Diseases.

Best regards,

Shaden Kamhawi

co-Editor-in-Chief

PLOS Neglected Tropical Diseases

Paul Brindley

co-Editor-in-Chief

PLOS Neglected Tropical Diseases
